# Occurrence of Deoxynivalenol in Maize and Wheat in Serbia

**DOI:** 10.3390/ijms9112114

**Published:** 2008-11-04

**Authors:** Igor Jajić, Verica Jurić, Dragan Glamočić, Biljana Abramović

**Affiliations:** 1 Faculty of Agriculture, Department of Animal Science, Trg D. Obradovića 8, 21000 Novi Sad, Serbia. E-Mails: jajic@polj.ns.ac.yu (I. J.); vjuric@polj.ns.ac.yu (V. J.); glamocic@polj.ns.ac.yu (D. G.); 2 Faculty of Science, Department of Chemistry, Trg D. Obradovića 3, 21000 Novi Sad, Serbia

**Keywords:** Deoxynivalenol, maize, wheat, HPLC, ELISA, Serbia

## Abstract

A total of 226 samples of maize and 59 of wheat from the 2004–2007 harvests were investigated for the presence and concentration of deoxynivalenol (DON). Samples of the 2004 harvest were analyzed after their storing for one year in barns, while those of the 2005–2007 harvest were taken directly off fields immediately after the harvest. The samples were analyzed by liquid chromatography on an ODS Hypersil column with DAD detector and ELISA methods. The average incidence rate of DON in maize from the 2004 harvest was 50% (concentration range 0.042–2.460 mg/kg, average value 0.536 mg/kg), while for those of the 2005–2007 harvest it was 32.4% (concentration range 0.027–2.210 mg/kg, average value 0.223 mg/kg). In the case of wheat incidence rate of DON for 2004 harvest was 50.0% (concentration range 0.630–1.840 mg/kg, average value 1.235 mg/kg), while for those of the 2005–2007 harvest it was 34.5% (concentration range 0.057–0.423 mg/kg, average value 0.190 mg/kg). Concentrations in two samples of maize and one of wheat (one sample of each cereal being of the 2004 harvest) were above the maximum level adopted by the European Commission. The results obtained were analyzed as a function of climatic conditions and compared with those of the neighboring countries where the relevant data existed.

## 1. Introduction

Food safety continues to be an important issue in the whole world. Each year, a large number of crops are affected by fungal invasions, leading to considerable financial losses and impaired health in animals and humans. Toxicity is mainly caused by secondary metabolites of fungi, which are appropriately called mycotoxins [1]. The most important agriculturally toxigenic fungi occurring in the moderate climatic zones of North America and Europe are *Fusarium* fungi [2]. Three of the more prevalent mycotoxins that occur in grain are deoxynivalenol (DON, vomitoxin), fumonisins and zearalenone [3].

DON is a member of the trichothecene family of mycotoxins. Structurally, it is a polar organic compound which belongs to the type B trichothecenes, and its chemical name is 12,13-epoxy-3α,7α,15-trihydroxytrichothec-9-ene-8-one. The occurrence of DON is associated primarily with *Fusarium graminearum* (teleomorph *Gibberella zeae*) and *Fusarium culmorum* (teleomorph unknown), both of which are important plant pathogens commonly found in cereals and other crops [4]. Although DON is among the least toxic of the trichothecenes, it is the most frequently detected one throughout the world, and its occurrence is considered to be an indicator of the possible presence of other, more toxic trichothecenes [5]. Consumption of contaminated feeds by livestock has been associated with a variety of adverse health effects including feed refusal (mainly by swine), reduced weight gain, diarrhea and emesis [6, 7].

Many international agencies are trying to achieve universal standardization of regulatory limits for mycotoxins. This is an incredibly difficult task as many factors have to be considered when deciding on regulatory standards. In addition to scientific factors, such as risk assessment and analytical accuracy, economical and political factors such as the commercial interests of each country and the constant necessity of a sufficient food supply also play a role in the decision making process. The Commission of the European Communities [8] established the following tolerance values for DON in cereals and cereal-based products: unprocessed cereals other than durum wheat, oats and maize (1250 μg/kg), unprocessed durum wheat and oats (1750 μg/kg), unprocessed maize (1750 μg/kg), cereal flour, including maize flour, maize grits and maize meal (750 μg/kg), bread, pastries, biscuits, cereal snacks and breakfast cereals (500 μg/kg), pasta (dry, 750 μg/kg), and processed cereal-based food for infants and young children and baby food (200 μg/kg). However, the maximum permitted level of DON in feed and groceries has not yet been set in Serbia. As for the products intended for animal feed are concerned, the European Commission guidance gives the following values for feeding stuffs with a moisture content of 12%: cereals and cereal products with the exception of maize by-products 8 mg/kg, maize by-products 12 mg/kg, complementary and complete feeding stuffs 5 mg/kg with the exception of complementary and complete feeding stuffs for pigs 0.9 mg/kg and complementary and complete feeding stuffs for calves (<4 months), lambs and kids 2 mg/kg [9]. In Serbia, the total permitted content of trichotecenes in feed for chickens, pigs (<50 kg) and calves is <300 μg/kg, while in feed for swine, ox and poultry it is <600 μg/kg [10].

Serbia is located in the moderate continental climate belt, where the most frequently isolated fungi contaminating cereals, feedstuffs, vegetables and fruits are from the *Fusarium, Penicillium* and *Aspergillus* genera [11]. The greatest outbreaks of epidemic moulds, mostly caused by *Fusarium* species, were registered in maize in 1955, 1968, 1972, 1974 and 1984, and in wheat in the early 1970s. The greatest outbreaks of animal diseases, especially diseases of pigs such as oestrogenism, vomiting and feed refusal, dermatoxicity, and others were recorded during the same and/or subsequent years. Fungi from the *Fusarium* genera are especially abundant, and with them *Fusarium* mycotoxins, first of all zearalenone, whose presence is mainly associated with *F. graminearum* and it is the highest in years with abundant precipitation and lower temperatures at the end of summer and the beginning of autumn [11]. Aflatoxins are rarely found in Serbia, but there is a possibility for a larger presence due to importation of soybean meal, earthnut cakes and others [12]. The natural occurrence of ochratoxin A is generally more important than the natural occurrence of aflatoxins under Serbian environmental conditions [11]. However, there are still no sufficient data about contamination of crops with DON in our country, although this mycotoxin could be expected because of the presence of moulds. Namely, in our previous preliminary investigations [13], we found the presence of DON in several crops (maize, wheat, soybean, sunflower and barley) in Serbia. It should be noted that the northern province of Serbia, Vojvodina, along with the neighboring countries Hungary, Croatia and Romania, is located in a region in which food production is one of the main industries. Wheat, primarily intended for human consumption, dominates in this region as a primary crop, along with maize, which represents the most important component of feed used in livestock nourishment. Serbia is a major regional producer of maize, ranking third in Central Europe behind Romania and Hungary [14].

Because of everything stated above, the primary aim of this study was to gain an insight into the presence of DON in Serbia on the basis of analysis of a larger number of samples of maize (226) and wheat (59) from the 2004–2007 harvests. Samples of the 2004 harvest were analyzed after their storing for one year in barns, while those of the 2005–2007 harvest were taken directly off fields immediately after the harvest. The samples were analyzed by liquid chromatography on an ODS Hypersil column with DAD detector, with the exception of maize samples of harvest 2007 which were analyzed by the ELISA test. Besides, since Serbia has similar climatic conditions as Croatia, Hungary, Romania and Bulgaria, and since the choice of crops, the use of agrotechnical measures and food production techniques are traditionally relatively similar, this paper attempts to compare data with those found in the relevant literature concerning the incidence of DON in this part of Southeast Europe.

## 2. Results and Discussion

Many analytical procedures have been developed for the determining DON in feed and food [15]. In this work the determination of DON in the collected maize and wheat samples was carried out by liquid chromatography under previously determined optimal experimental conditions [16], while maize samples of the 2007 harvest were analyzed by the ELISA test. Samples of the 2004 harvest were analyzed after their storing for one year in barns (Table 1), while those of the 2005–2007 harvest were taken directly off fields immediately after the harvest (Table 2). In 10 samples of maize and 4 of wheat from the harvest 2004 DON content above the limit of quantification value was found in 50%, and in one sample of each crop it exceeded the permitted values according to the EU Commission [8], i.e. 1.75 for maize and 1.25 mg/kg for wheat. However, it should be borne in mind that samples of the 2004 harvest were analyzed after their storing for one year in barns as well as that total number of was rather small, so that the obtained data cannot be interpreted as the actual situation in the fields in 2004. The number of samples collected and analyzed in 2005 (Table 2) was higher – 76 of maize and 12 of wheat, of which DON contamination was found with 42.1 and 33.3%, respectively. However, the DON content of 2.21 mg/kg in only one maize sample exceeded the tolerable level. As for the 2006 harvest, out of 21 maize and 34 wheat samples 38.1 and 35.3%, respectively were contaminated, and none of the samples of both crops contained DON above the EC permitted value. The number of analyzed samples of maize from the 2007 harvest was the largest – 119, but the number of wheat samples was only 9. Contamination of maize by DON was found with 25.2% and of wheat with 33.3%, and none of the analyzed samples had a DON content above the permitted value.

According to the literature data, the optimal temperature for *F. graminearum* growth is 25 °C, at a water activity above 0.88, while for growth of *F. culmorum* it is 21 °C, at a water activity above 0.87 [4]. The most favorable conditions for DON production in maize by *F. graminearum* according to Martins and Martins [17] are 22 °C (6.0 mg/kg) and 28 °C (5.5 mg/kg), after 35 days of incubation. These authors have also found that at the temperature of 37 °C *F. graminearum* did not produce DON. Besides, it is known that the incidence of Fusarium head blight is strongly associated with moisture at the time of flowering (anthesis), and the timing of rainfall, rather than its amount [4]. According to a recent study [18] on wheat, environment effects accounted for 48% of the variation in DON across all fields, followed by variety (27%), and previous crop (14 to 28%). Using actual data from farm fields, critical periods of weather were identified with DON concentrations in grain at maturity. Timing of those periods is relative to the average heading date (Zadoks 59) of the wheat crop. Three of those critical periods – from 4 to 7 days (1^st^ critical period) before heading and 3 to 10 days (2^nd^ and 3^rd^ critical periods) after heading – represent the most important contributors to the variation in DON. For wheat, in the climatic region of Serbia, this is the end of the month of May and the beginning of June. In case of maize, the infection of the ear most frequently takes place through the tip of the ear, when the fungi penetrate through the silk in the phase of maize flowering [19]. Exceptionally humid weather in the period from silking to ripening enables ear contamination [20]. The ear is the most sensitive to contamination at the beginning of silking, while this sensitivity lowers with silk aging [21,22]. The silking period in the climatic region of Serbia takes place within about 60 days from the moment of plant sprouting (during the month of July and the first half of August). According to the mentioned study [18] concerning maize, hybrid accounted for 25% of the variation of either DON or fumonisin, followed by environment (12%), and when combined 42% of the variability was accounted for. As can be seen, the influence of environment is much more pronounced with wheat than with maize.

**Figure 1.**
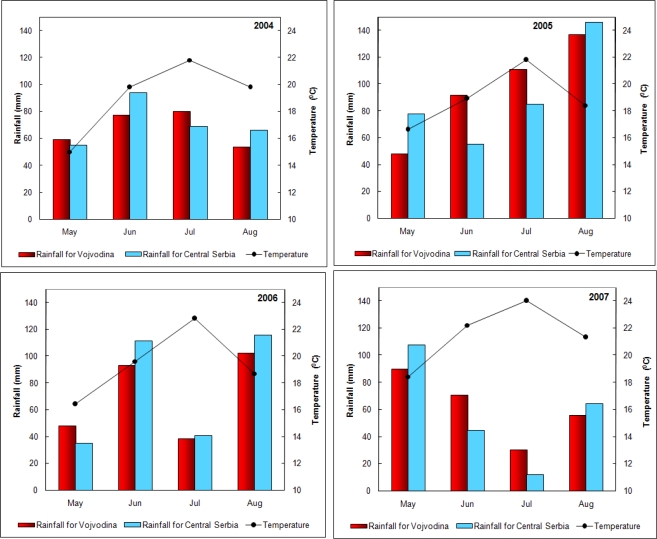
Monthly amounts of rainfall for the territories of Vojvodina and central Serbia and the mean monthly air temperatures in the same period for the territory of Serbia.

The climatic conditions for the period May–August for the above four years, as reported by the Republic Hydrometeorlogical Institute [23–26], are presented in Figure 1. The rainfall data are shown separately for Vojvodina (northern province of Serbia) and Central Serbia (the remaining part of Serbia excluding Kosovo and Metohija), as such data were available from the Republic Hydrometeorological Institute. However, it can be seen that the differences in the amount of precipitation were not significant. By comparing the data for May and June – the months that are of crucial importance for DON appearance in wheat, it can be seen that May of 2004 was the coldest (in the middle of the third decade the mean temperatures were in the range of 10–12 °C) and thus most unfavorable for the development of *Fusarium* fungi. However, the observed high proportion of wheat samples contaminated by DON (50%) in 2004 can be explained by the fact that the samples were analyzed after their storing for one year, so that the DON contamination was caused by the presence of moulds in the storage room and not in the field. As far as the 2005–2007 harvests are concerned the conditions for the DON occurrence were similar. Namely, the temperatures in May and June were favorable for the development of *Fusarium* fungi, whereas the rainfalls were in average similar, the only exception being May of 2007, when the humidity was much above the long-term mean. However, in view of the statement that the occurrence of rain is more significant than its amount [4] the DON occurrence as well as its amount in the 2005–2007 period were similar (Table 2).

When the climatic conditions in July and August are concerned, which are of crucial importance for DON occurrence in maize, it can be stated that all four years, with the exception of the year 2007 which was characterized by extremely high temperatures, were equally favorable for the growth of *F. graminearum,* and thus for the occurrence of DON. Namely, in the middle of July of 2007, very hot air from Africa penetrated the Balkan Peninsula, resulting in a number of days with tropical conditions with temperature maxima ≥30 °C, and the highest ever measured temperature (44.9 °C) in the Republic of Serbia. However, the amount of rainfall was below a multi-annual mean.

Similar weather conditions were also observed in August, when mean monthly temperatures were in the category of extremely high and well above the multi-annual mean. In this period, in the major part of Serbia there were 16 to 24 tropical days, which is twice more compared with a mean observed for many years. However, irrespective of the so high temperatures, the total number of rainy days remained at the level of the multi-year mean. Such dry summer with "tropical" temperatures and low humidity (July) was unfavorable for moulds development in the fields. The contamination of maize samples of 25.2%, with the maximal DON content of 0.172 mg/kg, may be considered even high. However, it should be pointed out that the samples were analyzed by the ELISA test, whose limit of determination is lower compared to HPLC. Besides, the results obtained by ELISA tests are slightly higher than those obtained by HPLC (Table 4). The weather conditions in July and August of 2004 could be considered as favorable for the occurrence of DON. Bearing in mind that the samples were analyzed after storing for one year and that DON occurrence was a results of the presence of moulds in the storage room, it is understandable that the contamination rate was high (50%) and one of the samples had the highest DON content measured in the four-year period (2.46 mg/kg). The period July–August of 2005 may be classified as ”extremely humid” and very favorable for DON occurrence. The contamination of maize samples was 42.1%, which is in accord with the weather conditions, and one maize sample had DON content of 2.21 mg/kg, which was above the permitted level. July of 2006 was characterized by a humidity which was below average value, whereas high humidity in August (rainy to extremely rainy category) undoubtedly contributed to contamination of maize samples (38.1%).

The results obtained in this study were compared with those from other countries of the world, including EU, and especially with those of the neighboring countries – Croatia, Hungary, Romania, and Bulgaria. However, the literature survey gave no data for DON occurrence in Macedonia, Bosnia and Herzegovina and Albania.

A study which encompassed a great number of countries, performed by JECFA [4], showed that DON was a frequent contaminant of cereal grains. Out of 11,444 analyzed wheat samples 57% were contaminated by DON (concentration range 1–5,700 μg/kg), and of 5349 maize samples DON was present in 40% (concentration range 3–3,700 μg/kg). A European study [27] on occurrence of *Fusarium* toxins revealed that 57% of the samples from 11 countries (11,022 samples) were positive for DON. A high frequency of DON was found in maize (89%) and wheat and wheat flour (61%).

In Hungary, Rafai *et al.* [28] analyzed DON content in samples of maize, wheat soybean, barley, bran, tritical, oats, rye and sunflower, harvests 1991–1998. These authors found high contamination rate of wheat (78.2%), and surprisingly low of maize (10.8%). Such difference in the DON contamination they explained by agricultural factors and different sensitivity to the presence of *Fusarium* moulds. Unfortunately, although the authors presented results for a longer time period, they did not give data about climatological conditions and potential relationship between climatic factors and toxin occurrence. In a study of the DON presence in wheat samples of the 1998 harvest, Fazekas *et al.* [29] found that 88% of them were contaminated by DON. This, much higher percentage of wheat contamination by DON compared to the usual levels, the authors related to the extremely rainy summer.

Sokolović and Šimpraga [30] analyzed DON in cereal grains (14) and feed (37) in Croatia in samples collected in the 2001–2004 period using thin layer chromatography. The rate of cereal grain samples positive for DON was 28.6% and for feed 45.9%, with the concentration range of 0.1 3.4 mg/kg and 0.05 1.05 mg/kg, respectively. But, according to the authors, the number of analyzed samples was small, and could not be interpreted as the actual situation in the field conditions. As far as climatic conditions are concerned, the period from 2001–2004 was extremely warm and the years 2003 and 2004 were highly humid. Although the authors state that the climatic conditions for the said period were similar, especially for 2003 and 2004, partial results for incidence rate of DON for individual years are still very different (i.e. grain 33.3 and 71.4%, respectively).

By investigating 140 wheat samples from the 1995 harvest by immunochemical methods, for the presence of *Fusarium* mycotoxins in Bulgaria [31] it was found that DON and zearalenone are dominant, being present with 67 and 69% respectively. The average level of DON in positive samples was 0.180 mg/kg. The authors state that regarding the climatic conditions, heavy rainfall were observed in all regions of the country in the spring and summer months of 1995.

Curtui *et al.* [32] analyzed samples of wheat (25) and maize (30), collected in 1997 after harvest from western Romania, by enzyme immunoassays. Frequency of DON contamination was 100% in wheat samples (median value at 0.880 mg/kg and maximum concentration 5.600 mg/kg), and 46% in maize samples (median value 0.890 mg/kg and maximum concentration 160.0 mg/kg). Climatic conditions prevailing in the summer months of 1997 were characterized by heavy rainfall before the harvest.

Although the study by Rafai *et al.* [28], in view of the crop type and number of samples, as well as the time period encompassed, were most detailed compared to the other investigations in the neighboring countries, the authors missed to supply data about pertaining climatic conditions. In respect of the obtained results about DON presence in crops in the region, it can be concluded that they are much diversified. Namely, significant differences can be seen in the results obtained by analyzing maize samples in Hungary [28], with a low contamination rate by DON (10.8%) compared to those in Romania and Serbia (46 and 33%, respectively). Similar results (40% positive samples) were reported by the JECFA [4], whereas European Commission in its report [27] quoted significantly higher contamination rate of maize, even 89%. The DON contamination of wheat samples collected in Serbia (36%) was the lowest compared to the above data. Only Sokolović and Šimpraga [30] reported lower values (28.6%), but they did not mention that wheat was in question, as they used the term cereals.

The above differences in the contamination levels may be related to the agricultural factors and partly to the different sensitivity of crops to *Fusarium* species in the interaction with climatic conditions. Also, it is necessary to take into account analytical methods used for DON analysis, as they have different limits of detection, giving thus the different percent of contaminated samples. This is especially important when one compares results with those presented in the JECFA [4] and EC [27] studies.

## 3. Experimental Section

### 3.1. Chemicals

All solvents used for the DON extraction from cereal samples, as well as for the mobile phase preparation, were of HPLC grade. All chemicals used in the investigation were of reagent grade. Solutions were prepared in doubly deionized water except when stated otherwise.

#### DON standard solutions

DON (Biopure, Tulln, Austria) was purchased as an analytical standard. Calibrant solution was prepared in ethyl acetate–methanol (19:1, v/v) at the concentration of 0.1–0.2 mg/ml from crystalline substance according to AOAC method 986.17. Stock solution was prepared by measuring 1.00 mL of calibrant solution of DON into a 5 or 10 mL volumetric flask and diluting to volume with ethyl acetate–methanol (19:1, v/v). Working calibrant solutions were prepared by evaporating the appropriate volume of the stock solution and diluting with 1.00 mL of methanol. Standard solutions were stored at 4 °C.

### 3.2. Samples

Samples of cereals from the 2004–2007 harvests were collected from different locations in the Republic of Serbia. Samples of the 2004 harvest were taken from barns, i.e., storage sites, where they had been kept for one year, while those of the 2005–2007 harvest were taken directly off fields immediately after the harvest. Immediately after sampling, 1000 g of each sample were prepared by grinding in a laboratory mill in such a way that >93% passed through a sieve with pore diameter of 0.8 mm. After that, the sample was homogenized by mixing. Samples thus prepared were packed in plastic bags and stored in a freezer at −20 °C until analysis. Prior to each analysis, the samples were allowed to reach room temperature.

### 3.3. HPLC determination

#### Extraction and clean-up

An activated charcoal–alumina–Celite–cation exchange resin column [16] was used for purification. The samples (25.0 g) were extracted with acetonitrile–water (84:16, v/v, 100 mL) and shaken on a magnetic stirrer for 60 min. After filtration through Advantec filter paper, an aliquot of the extract (6.0 mL) was applied to the prepared column. The column was then washed with a solvent mixture (5 mL) comprised of acetonitrile–water (84:16, v/v) at a rate of about 0.6 mL/min. The cleaned-up extract was evaporated to dryness, dissolved in ethyl acetate (3 mL) and quantitatively transferred to an evaporation vessel by triple washing with ethyl acetate (1.5 mL). The eluate was evaporated just to dryness.

#### Liquid chromatographic analysis

The equipment consisted of an LC system – HP 1090 Liquid Chromatograph (Hewlett Packard, Palo Alto, CA, USA) with a DAD detector (Hewlett Packard, Palo Alto, CA, USA) and a column Hypersil ODS (100 × 4.6 mm i.d., particle size 5 μm, Agilent Technologies, USA). LC analysis of DON was performed after evaporation, the residue was redissolved in methanol (300 μL), and an aliquot of the solution (15 μL) was injected into the LC system. The mobile phase consisting of a mixture of acetonitrile–water (16:84, v/v) was used at 0.6 mL/min. UV detection was performed at 220 nm. The mobile phase was filtered through a 0.45 μm membrane (Aura industries, TFM, Hewlett Packard).

#### Analytical quality control

Calibration curves used for the quantitative determination were constructed on the basis of the area under the DON chromatographic peaks, using seven DON working standard solutions. The linearity of the method was assessed by standards ranging from 0.17–3.40 ng/μl. The correlation coefficient was 0.999. The limit of quantification for LC determination based on a signal-to-noise ratio of 10:1 was 0.20 ng/μL of DON, which is equivalent to 0.040 mg/kg of DON in substrate. Recovery studies were performed on the blank samples of each cereal spiked with levels of 0.70 and 1.10 mg/kg of DON (Table 3). The results for the samples were not corrected for the recovery of the spike.

### 3.4. Immunochemical determination

#### Extraction

The procedure of sample preparation for immunochemical determinations was identical to that for HPLC, the only difference being that 20 g of samples were weighed in a 200 mL beaker. DON was extracted with distilled water (100 mL) on an Ultra Turax T18 homogenizer for 3 min at 11,000 rpm. Crude extract was then filtered through 5B Advantec filter paper.

#### Analysis

The immunochemical analysis was performed using the Veratox, DON HS (quantitative high sensitivity test) (Neogen, Lansing, MI, USA). In the mixing well were placed the conjugate (100 μL) and the standard or sample (100 μL). To make a homogeneous mixture, the liquid was drawn up and dispensed three times with the aid of a micropipette. A volume of the mixed solution (100 μL) was transferred to the well with antibodies and incubated at room temperature for 10 min. The solution was then removed and the well was washed 5 times with distilled water. After that the substrate (100 μL) was added and 10–min incubation was stopped by adding "stop" reagent (100 μL). Optical densities on the basis of which DON content was calculated were read using the reader of microtitration plates with a 630 nm filter. According to the manufacturer the limit of determination was 0.025 mg DON/kg sample, and range of quantitation 25–250 ppb.

As can be concluded from the literature data, ELISA tests give results that are higher than the real ones [33], which is explained by the reaction of antibodies with other trichothecenes of group B too. Hence a number of maize samples were analyzed by both methods. As can be seen from Table 4, ELISA tests showed DON contamination with 12 samples, whereas HPLC analysis of the same samples showed it only in 5 cases. This difference is a consequence of the lower limit of determination of ELISA tests. It can also be seen that the results obtained by immunochemical method are higher than those measured by HPLC method, which is in accordance with the literature data.

## 4. Conclusions

Summing up the results obtained for the three-year period (2005–2007) when samples were taken directly off fields immediately after the harvest it may be stated that out of the analyzed 216 maize samples DON was registered in 32.4% of them, maximum content being 2.21 mg/kg. In the 55 wheat samples collected in the same period the DON contamination was found in 34.5% of cases, and the highest DON content was 0.423 mg/kg. Concentration range of DON was either low or medium. Of all the samples analyzed, only one sample of maize contained DON in an amount that was higher than the permitted EC levels. By comparing our results with data in the relevant literature, it can be said that although incidence rate of DON in Serbia-grown cereals was occasionally considerable, the position of the country is not worse than the average of the surrounding countries. By regulating the maximum permitted level of DON in feed and food in Serbia, as well as by establishing monitoring programs, the risk to the consumer could be minimized. It should be noticed that the percentage of contaminated samples and DON content in samples of the 2004 harvest, which were analyzed after their storing for one year in barns, were higher although the weather conditions were even less favorable for DON occurrence, which indicates that the way of storing is also a very important factor in the occurrence of DON.

## Figures and Tables

**Table 1.** Occurrence of DON in maize and wheat in Serbia from the 2004 harvest.

Cereal	Year	No. of samples	No. of positive samples (%)	Concentration in samples		
				Average±SD (mg/kg)	Range (mg/kg)	Median (mg/kg)
Maize	2004	10	5 (50.0)	0.536±1.076	0.042–2.460	0.050
Wheat	2004	4	2 (50.0)	1.235±0.856	0.630–1.840	1.235

**Table 2.** Occurrence of DON in maize and wheat in Serbia from the 2005–2007 harvests.

Cereal	Year	No. of samples	No. of positive samples (%)	Concentration in samples		
				Average±SD (mg/kg)	Range (mg/kg)	Median (mg/kg)
Maize	2005	76	32 (42.1)	0.327±0.392	0.040–2.210	0.229
	2006	21	8 (38.1)	0.426±0.396	0.140–1.340	0.260
	2007	119	30 (25.2)	0.058±0.039	0.027–0.172	0.040
	**∑ 2005 2007**	**216**	**70 (32.4)**	**0.223**±**0.328**	**0.027–2.210**	**0.111**

Wheat	2005	12	4 (33.3)	0.182±0.171	0.057–0.423	0.124
	2006	34	12 (35.3)	0.223±0.075	0.090–0.410	0.223
	2007	9	3 (33.3)	0.177±0.033	0.142–0.208	0.182
	**∑ 2005 2007**	**55**	**19 (34.5)**	**0.190**±**0.111**	**0.057–0.423**	**0.182**

**Table 3.** Recovery and precision of DON assays run on analyzed cereals (n = 3).

Cereal	Added (mg/kg)			
	0.70		1.10	
	Recovery (%)	SD	Recovery (%)	SD
Maize	97.1	2.9	95.2	1.5
Wheat	90.9	2.3	90.2	1.9

**Table 4.** Comparison of data for DON obtained for maize samples by ELISA and HPLC methods.

Sample	ELISA *c*(mg/kg)	HPLC *c*(mg/kg)
1	0.029	ND
2	0.028	ND
3	0.059	ND
4	0.036	ND
5	0.062	ND
6	0.098	0.067
7	0.035	ND
8	0.166	0.110
9	0.072	ND
10	0.095	0.078
11	0.173	0.144
12	0.092	0.073
13–20	–	–
